# Supplementary material for: Evaluation of internal coupling and coordination degree and diagnosis of obstacle factors for high-quality regional economic development: Evidence from Chongqing’s " One District, Two Groups"
Source: PLoS One. 2024 Dec 20;19(12):e0312820. doi: 10.1371/journal.pone.0312820 (PMC11661609; doi:10.1371/journal.pone.0312820)
Supplement: Attachment — Submitted filename: Response to Reviewers.docx [file pone.0312820.s002.docx]

**Response to Reviewers**

Dear reviewers:

Greetings!

Thank you very much for your meticulous review and professional comments! The problems and suggestions you have pointed out are very targeted and constructive, and the author has benefited a lot from them. Based on the review comments, the author has conducted more in-depth thinking and research on related issues, and then revised and improved the article. The following is a detailed response to your review comments, and the corresponding changes are reflected in the revised text in the revised mode.

Detailed revisions to the reviewer's comments on the article are described below:

# Reviewer #1:

## 1.Why the index system in Table 1 can be used to evaluate high-quality economic development?

Currently, the index system is mainly constructed based on researchers' understanding of the connotation of high-quality economic development, and some scholars have constructed a high-quality economic development evaluation index system in accordance with the “five new development concepts” and sustainable development (Arrow K J et al.,2012;Chen L,2022;Yang Y et al. 2024), which meets the requirements of economic development in the new era, but needs to be improved to reflect the actual high-quality economic development, which fits the requirements of economic development in the new era, but there is still room for improvement in reflecting the actual high-quality development of the economy; and some researchers measure high-quality development of the economy from the level of efficiency, for example, some studies have used labor productivity (Chen S et al., 2018;Chen Shiyi et al, 2018) , total factor productivity (Changzheng Z, 2010; Liu Zhibiao et al, 2020；Hua X, 2021) or GDP per capita(Ge H, 2022) to measure the high quality of the economy, and these indicators can effectively characterize the level of high-quality economic development from a certain perspective, but they are still not comprehensive enough.

## 2.The literature used in literature review is too old. & A large number of references are not searchable in the web of science, which I think is unacceptable.

Since the research content of this paper is based on China's judgment of its own economic development stage and analyzes the regional division of Chongqing, China's “one district and two groups”, most of the references come from the results of China's domestic research on high-quality economic development in the past five years. Therefore, it is difficult to find the relevant literature in the English journal website. Since China's economic high-quality development was formally proposed in 2017, some of the literature on the definition of economic high-quality development cited in the article exists with an earlier publication date. Meanwhile, the paper also introduces some recent literature in the indicator description section, which can be found in "Reviewer #2: No. 5".

In this paper, we add the following to the literature review section: With the depth of research, scholars have found that innovation-driven (Du J, 2020; Zhou C, 2022; Guo B, 2023), green finance (Yang Y, 2021; Gao J, 2023), digital economy (Zhang W, 2021; Guo B, 2023), environmental regulation (Chen L, 2020; Wang J, 2022) and other factors can promote high-quality economic development, while resource dependence can seriously hinder high-quality economic development (Du J, 2020). Therefore, these factors should also be taken into consideration when designating the evaluation index system for high-quality economic development.

# Reviewer #2:

## 1.It is suggested to briefly explain why Chongqing's "one district and two groups" was chosen as the research subject, highlighting the uniqueness and importance of this strategy for the harmonious development of Chongqing's regional economy.

Chongqing’s “one district and two groups” was chosen as the object of study because of the uniqueness and importance of this strategy for the harmonious development of Chongqing’s regional economy. As an important city in the development of western China, Chongqing’s “one district and two groups” strategy aims to optimize the regional economic structure, promote regional integration, and achieve efficient allocation of resources and balanced economic development.

The “one district” refers to the main urban area of Chongqing, which is the political, economic and cultural center of Chongqing and has a strong radiation and driving effect on the development of the surrounding areas. By improving the core competitiveness of the main city, it can effectively promote the regional economic agglomeration and diffusion effect. The “two groups” refer to the town clusters in the Three Gorges Reservoir Area in northeast Chongqing and the town clusters in the Wuling Mountain Area in southeast Chongqing, which are tasked with promoting local economic development while protecting the ecological environment. Through the development of these two regions, the industrial division of labor and complementary advantages within the Chongqing region can be realized, and the balanced development of the regional economy can be promoted. The implementation of this strategy will help to play the role of radiation driven by the main city of Chongqing, promote the economic development of the surrounding areas, but also help to alleviate the pressure of the development of the main city, to achieve balanced regional development.

In addition, Chongqing's “one district and two groups” strategy is also closely related to the construction of the Chengdu-Chongqing Twin-city Economic Circle. The Chengdu-Chongqing Twin Cities Economic Circle is an important economic growth pole in western China, and its development is of great significance in promoting the economic development of western China. Through the strategy of “one district and two groups”, Chongqing can strengthen cooperation with neighboring cities such as Chengdu, jointly promote the harmonious development of the regional economy and form new economic growth points.

Chongqing's “one region, two groups” strategy also focuses on green and innovative development, which will help promote high-quality economic development. By optimizing the industrial structure and developing high-tech industries and modern service industries, Chongqing can achieve sustainable economic development while protecting the ecological environment.

In summary, Chongqing’s “one region, two groups” strategy is not only an important measure to promote the harmonious development of the regional economy, but also an effective way to achieve high-quality economic development. The implementation of this strategy will help elevate Chongqing's position in the overall development of the country and inject new vitality into the economic development of western China.

## 2.The analysis of obstacle factors currently only covers the impact of primary indicators. It is recommended to refine the analysis further by examining specific deficiencies in policies, infrastructure, human resource development, technological innovation, and other relevant aspects through an in-depth analysis of specific indicators.

## 4.4 Diagnosis and analysis of obstacle factors

On the basis of the overall evaluation mentioned above, we diagnose the obstacle factors affecting the high-quality development of Chongqing's economy, and identify the main obstacle factors according to the criterion-level indicators and indicator-level indicators, and the results are shown in Tables 7-10.

## 4.4.1 Guideline level analysis

**Table 7. Results of high-quality development barriers at the guideline level in Chongqing from 2017-2021.**

| **Subject investigated** | **Economic vigor** | **Harmonious development** | **Green development** | **Digital development** |
| --- | --- | --- | --- | --- |
| **In 2017, the barrier degree mean value** | 0.5394 | 0.2344 | 0.2037 | 0.0224 |
| **In 2018, the barrier degree mean value** | 0.5366 | 0.2312 | 0.1947 | 0.0374 |
| **In 2019, the barrier degree mean value** | 0.5484 | 0.2693 | 0.1562 | 0.0261 |
| **In 2020, the barrier degree mean value** | 0.5493 | 0.2613 | 0.1657 | 0.0237 |
| **In 2021, the barrier degree mean value** | 0.5479 | 0.2664 | 0.1601 | 0.0256 |

From Table 7, it can be found that during the five-year period of 2017-2021, the ranking of obstacle indicators of each criterion layer is “economic vigor > harmonious development > green development > digital development”, and the higher obstacle degree means that the heavier the impact of the indicator, indicating that economic vigor has a very important position in the high-quality development of Chongqing's economy.

**Table 8. Results of high-quality development barriers at the guideline level in "One District and two Groups" from 2017-2021.**

| **Year** | **Area** | **Economic vigor** | **Harmonious development** | **Green development** | **Digital development** |
| --- | --- | --- | --- | --- | --- |
| **2017** | Main city metropolitan area | 0.2294 | 0.2071 | 0.5635 | 0 |
| **2017** | Northeast Chongqing | 0.6964 | 0.2534 | 0.0229 | 0.0273 |
| **2017** | Southeast Chongqing | 0.6925 | 0.2428 | 0.0247 | 0.0400 |
| **2018** | Main city metropolitan area | 0.2416 | 0.1993 | 0.5101 | 0.0490 |
| **2018** | Northeast Chongqing | 0.6635 | 0.2407 | 0.0587 | 0.0370 |
| **2018** | Southeast Chongqing | 0.7048 | 0.2536 | 0.0154 | 0.0263 |
| **2019** | Main city metropolitan area | 0.2024 | 0.3922 | 0.4055 | 0 |
| **2019** | Northeast Chongqing | 0.6814 | 0.2428 | 0.0390 | 0.0369 |
| **2019** | Southeast Chongqing | 0.7615 | 0.1729 | 0.0242 | 0.0414 |
| **2020** | Main city metropolitan area | 0.2173 | 0.3560 | 0.4267 | 0 |
| **2020** | Northeast Chongqing | 0.6718 | 0.2438 | 0.0461 | 0.0383 |
| **2020** | Southeast Chongqing | 0.7586 | 0.1843 | 0.0244 | 0.0327 |
| **2021** | Main city metropolitan area | 0.2103 | 0.3701 | 0.4196 | 0 |
| **2021** | Northeast Chongqing | 0.6837 | 0.2547 | 0.0229 | 0.0387 |
| **2021** | Southeast Chongqing | 0.7498 | 0.1745 | 0.0377 | 0.0380 |

From Table 8, it can be found that during the period of 2017-2021, the results of obstacles to high-quality economic development in the guideline level of southeast Chongqing and northeast Chongqing are consistent with the overall situation of Chongqing, i.e., “economic vigor > harmonious development > green development > digital development”, which indicates that the key factor hindering the development of the “two groups” is still economic vigor, followed by harmonious development, and green development is the third. This indicates that the key factor hindering the development of the “two groups” is still economic vigor, followed by harmonious development and green development. However, in the main city metropolitan area, it is “green development > economic vigor > harmonious development > digital development”, which means that the green development indicator is the key factor hindering the development of the main city metropolitan area, followed by economic vigor, and third by harmonious development indicator. Meanwhile, it is worth noting that the degree of obstacles to digital development in the main urban metropolitan area is 0 in the five-year period from 2017 to 2021, which indicates that the digital development of the main urban metropolitan area is at a high level, and it will not constrain the high-quality development of the economy of the main urban metropolitan area.

## 4.4.2 Indicator level analysis

Due to the large number of indicators in the indicator layer, according to the size of the obstacle degree, this paper only lists the top seven factors in the ranking of obstacle degree, and the results are shown in Table 9.

**Table 9. Results of high-quality development barriers at the indicator level in Chongqing from 2017-2021.**

| **Year** | **Event** | **Ranking of barrier factors** | | | | | | |
| --- | --- | --- | --- | --- | --- | --- | --- | --- |
|  |  | **1** | **2** | **3** | **4** | **5** | **6** | **7** |
| **2017** | Barrier factors | Density of population | The added value of industry | Groundwater resources amount | Foreign trade dependence degree | The proportion of financial loan balance | Patent authorization | Advanced industry |
|  | Barriers | 0.1307 | 0.1029 | 0.1025 | 0.0804 | 0.0539 | 0.0538 | 0.0451 |
| **2018** | Barrier factors | Density of population | The added value of industry | Groundwater resources amount | Foreign trade dependence degree | The proportion of financial loan balance | Patent authorization | Advanced industry |
|  | Barriers | 0.1285 | 0.1012 | 0.1004 | 0.0801 | 0.0548 | 0.0538 | 0.0451 |
| **2019** | Barrier factors | Density of population | The added value of industry | Advanced industry | Groundwater resources amount | Foreign trade dependence degree | Patent authorization | The proportion of financial loan balance |
|  | Barriers | 0.1340 | 0.1104 | 0.0826 | 0.0825 | 0.0811 | 0.0558 | 0.0539 |
| **2020** | Barrier factors | Density of population | The added value of industry | Groundwater resources amount | Foreign trade dependence degree | Advanced industry | The proportion of financial loan balance | Patent authorization |
|  | Barriers | 0.1350 | 0.1109 | 0.0867 | 0.0772 | 0.0725 | 0.0574 | 0.0546 |
| **2021** | Barrier factors | Density of population | The added value of industry | Groundwater resources amount | Advanced industry | Foreign trade dependence degree | Patent authorization | The proportion of financial loan balance |
|  | Barriers | 0.1358 | 0.1117 | 0.0815 | 0.0779 | 0.0759 | 0.0557 | 0.0533 |

From Table 9, it can be found that during the period of 2017-2021, although there are changes in the ranking results of the barrier degree of the indicator layer in Chongqing, it is always the seven barrier factors of density of population, the added value of industry, groundwater resources amount, foreign trade dependence degree, the proportion of financial loan balance, patent authorization and advanced industry, which are mainly reflective of the development of the three dimensions of economic vigor, harmonious development, and green development. Specifically, as a populous city, the population density of Chongqing directly affects urban planning, infrastructure construction and public service provision, which in turn affects the stable and efficient operation of the economy; industry is an important pillar of Chongqing's economy, and the growth of the value added of industry directly reflects the efficiency of industrial production and the degree of optimization of industrial structure, which is fundamental to the high-quality development of the economy; water resources are one of the key factors constraining the economic and social development of the region; and water resources are one of the key factors constraining the economic and social development of the region. Water resources are one of the key factors constraining the economic and social development of the region. The amount of underground water resources in Chongqing is directly related to the safety of urban water supply, industrial and agricultural water demand and ecological environmental protection; Chongqing as an inland open highland, the dependence on foreign trade reflects the degree of openness and international competitiveness of its economy, and has an important impact on the diversification and stability of the economy; finance is the core of the modern economy, and the ratio of the balance of financial loans reflects the strength of financial support to the real economy, which is crucial for promoting industrial upgrading and economic restructuring. industrial upgrading and economic restructuring; the number of patents granted is an important indicator of innovation capacity, reflecting the level of scientific and technological innovation and industrial competitiveness of a region, which plays an important role in promoting high-quality development of the economy; industrial advancement represents the optimization and upgrading of the industrial structure, and it is an important symbol of high-quality development of the economy, which involves the modernization, high-endization and intelligence of the industrial chain.

**Table 10. Results of high-quality development barriers at the indicator level in "One District and two Groups" from 2017-2021.**

| **Year** | **Region** | **Event** | **Ranking of barrier factors** | | | | | | |
| --- | --- | --- | --- | --- | --- | --- | --- | --- | --- |
|  |  |  | **1** | **2** | **3** | **4** | **5** | **6** | **7** |
| **2017** | Main city metropolitan area | Barrier factors | Groundwater resources amount | Yearly precipitation | Asset-liability ratio | Per capita disposable income ratio of urban and rural residents | Land area covered with trees | All-personnel labour productivity | The proportion of education expenditure |
|  |  | Barriers | 0.3015 | 0.0990 | 0.0926 | 0.0897 | 0.0765 | 0.0760 | 0.0740 |
| **2017** | Northeast Chongqing | Barrier factors | Density of population | The added value of industry | Foreign trade dependence degree | The proportion of financial loan balance | Patent authorization | Advanced industry | R&D funding investment intensity |
|  |  | Barriers | 0.1860 | 0.1388 | 0.1321 | 0.0990 | 0.0723 | 0.0638 | 0.0493 |
| **2017** | Southeast Chongqing | Barrier factors | Density of population | The added value of industry | Foreign trade dependence degree | Patent authorization | Advanced industry | The proportion of financial loan balance | The proportion of financial deposit balance |
|  |  | Barriers | 0.2060 | 0.1698 | 0.1089 | 0.0891 | 0.0715 | 0.0627 | 0.0587 |
| **2018** | Main city metropolitan area | Barrier factors | Groundwater resources amount | All-personnel labour productivity | Yearly precipitation | Per capita disposable income ratio of urban and rural residents | Asset-liability ratio | Land area covered with trees | The proportion of education expenditure |
|  |  | Barriers | 0.2707 | 0.1075 | 0.0889 | 0.0805 | 0.0795 | 0.0687 | 0.0664 |
| **2018** | Northeast Chongqing | Barrier factors | Density of population | The added value of industry | Foreign trade dependence degree | The proportion of financial loan balance | Patent authorization | Advanced industry | R&D funding investment intensity |
|  |  | Barriers | 0.1777 | 0.1322 | 0.1263 | 0.0946 | 0.0715 | 0.0631 | 0.0478 |
| **2018** | Southeast Chongqing | Barrier factors | Density of population | The added value of industry | Foreign trade dependence degree | Patent authorization | Advanced industry | The proportion of financial loan balance | The proportion of financial deposit balance |
|  |  | Barriers | 0.2078 | 0.1713 | 0.1139 | 0.0899 | 0.0721 | 0.0697 | 0.0592 |
| **2019** | Main city metropolitan area | Barrier factors | Groundwater resources amount | Advanced industry | All-personnel labour productivity | Per capita disposable income ratio of urban and rural residents | Asset-liability ratio | Yearly precipitation | Land area covered with trees |
|  |  | Barriers | 0.2397 | 0.1831 | 0.0841 | 0.0713 | 0.0699 | 0.0631 | 0.0608 |
| **2019** | Northeast Chongqing | Barrier factors | Density of population | The added value of industry | Foreign trade dependence degree | The proportion of financial loan balance | Patent authorization | Advanced industry | R&D funding investment intensity |
|  |  | Barriers | 0.1774 | 0.1462 | 0.1262 | 0.0945 | 0.0703 | 0.0647 | 0.0478 |
| **2019** | Southeast Chongqing | Barrier factors | Density of population | The added value of industry | Foreign trade dependence degree | Patent authorization | The proportion of financial loan balance | The proportion of financial deposit balance | R&D funding investment intensity |
|  |  | Barriers | 0.2244 | 0.1850 | 0.1170 | 0.0971 | 0.0672 | 0.0639 | 0.0566 |
| **2020** | Main city metropolitan area | Barrier factors | Groundwater resources amount | Advanced industry | All-personnel labour productivity | Yearly precipitation | Per capita disposable income ratio of urban and rural residents | Asset-liability ratio | Land area covered with trees |
|  |  | Barriers | 0.2435 | 0.1530 | 0.0967 | 0.0800 | 0.0724 | 0.0715 | 0.0618 |
| **2020** | Northeast Chongqing | Barrier factors | Density of population | The added value of industry | Foreign trade dependence degree | The proportion of financial loan balance | Patent authorization | Advanced industry | R&D funding investment intensity |
|  |  | Barriers | 0.1783 | 0.1459 | 0.1261 | 0.0944 | 0.0658 | 0.0646 | 0.0462 |
| **2020** | Southeast Chongqing | Barrier factors | Density of population | The added value of industry | Foreign trade dependence degree | Patent authorization | The proportion of financial loan balance | The proportion of financial deposit balance | R&D funding investment intensity |
|  |  | Barriers | 0.2266 | 0.1868 | 0.1055 | 0.0980 | 0.0778 | 0.0645 | 0.0581 |
| **2021** | Main city metropolitan area | Barrier factors | Groundwater resources amount | Advanced industry | All-personnel labour productivity | Yearly precipitation | Asset-liability ratio | Per capita disposable income ratio of urban and rural residents | Land area covered with trees |
|  |  | Barriers | 0.2394 | 0.1683 | 0.0906 | 0.0786 | 0.0714 | 0.0712 | 0.0607 |
| **2021** | Northeast Chongqing | Barrier factors | Density of population | The added value of industry | Foreign trade dependence degree | The proportion of financial loan balance | Patent authorization | Advanced industry | R&D funding investment intensity |
|  |  | Barriers | 0.1801 | 0.1478 | 0.1273 | 0.0954 | 0.0687 | 0.0653 | 0.0462 |
| **2021** | Southeast Chongqing | Barrier factors | Density of population | The added value of industry | Foreign trade dependence degree | Patent authorization | The proportion of financial deposit balance | The proportion of financial loan balance | R&D funding investment intensity |
|  |  | Barriers | 0.2272 | 0.1873 | 0.1003 | 0.0983 | 0.0647 | 0.0646 | 0.0582 |

From Table 10, it can be found that during the period of 2017-2021, the top seven obstacles to the high-quality development of the economy of the northeast Chongqing include density of population, the added value of industry, foreign trade dependence degree, the proportion of financial loan balance, patent authorization, advanced industry and R&D funding investment intensity. During 2017-2018, the top seven obstacle factors affecting the high-quality development of the economy of the southeast Chongqing include density of population, the added value of industry, foreign trade dependence degree, patent authorization, advanced industry, the proportion of financial loan balance	, and the proportion of financial deposit balance, while after 2018, advanced industry exits the top seven and is replaced by R&D funding investment intensity. Although there are differences in specific obstacle factors, the results of the obstacle degree in the indicator layer of high-quality economic development in southeast Chongqing and northeast Chongqing are mainly reflected in the dimensions of economic vigor and harmonious development, indicating that the key factors for the development of the “two groups” are still economic vigor and harmonious development. It is worth noting that during the period of 2017-2021, the total proportion of population density and industrial value added obstacles in the obstacle factors of the two groups exceeds 30%, indicating that population density and industrial value added are the main constraints to the high-quality economic development of the two groups. This is because the lower population density in the “two groups” region leads to limited market size, insufficient consumer demand and labor supply, thus restricting the high-quality development of the economy. At the same time, the weak industrial base and low value-added of industry in the northeastern Chongqing and southeastern Chongqing make it difficult to drive the rapid growth of the regional economy and optimize and upgrade the industrial structure, thus affecting the high-quality development of the economy. During the period 2017-2021, the top seven barriers to high-quality economic development in the main city metropolitan area are still mainly reflecting the three dimensions of economic vigor, harmonious development and green development, although there are changes in the top seven barriers.

## 3.The conclusion could more clearly summarize the research findings and indicate the direction for future studies. It should provide an overview of the main research outcomes, the current state of regional economic development, and possible future improvement paths.

This paper measures and analyzes the internal coupling coordination level and obstacle factors of the high-quality economic development in Chongqing’s “one district and two groups.” The research builds a regional economic development evaluation system with 34 indicators, covering dimensions such as economic vitality, coordinated development, green development, and digital development. Using the entropy weight TOPSIS method, the study assesses the economic development level of Chongqing’s “one district and two groups” in the new era and applies the coupling coordination model and barrier factor model to analyze the internal coupling coordination development and identify the obstacle factors restricting high-quality economic development. The research findings indicate: (1)From 2017 to 2021, the high-quality economic development level of Chongqing’s “one district and two groups” increased yearly, driven by the joint growth of all dimensions. The main urban area’s development index was the highest, with the Wuling Mountain area in southeast Chongqing slightly surpassing the Three Gorges Reservoir area in northeast Chongqing. The growth rates showed that the main city new area developed fastest, followed by southeast Chongqing, northeast Chongqing, and finally the central city. While economic vitality, coordinated development, green development, and digital development levels increased, regional differences remained in various dimensions. (2)The internal coupling and coordination degree of high-quality economic development in Chongqing’s “one district and two groups” also improved yearly. The metropolitan area’s level far exceeded that of southeast and northeast Chongqing, with southeast Chongqing slightly ahead of northeast Chongqing. From 2017 to 2021, the main city metropolitan area progressed from forced coordination to good coordination, northeast Chongqing from serious imbalance to primary coordination, and southeast Chongqing from moderate imbalance to primary coordination, indicating a gradual formation of internal coupling and coordinated development. However, convergence analysis showed that the coupling coordination degree of high-quality economic development in the “one district and two groups” diverged, indicating a widening trend rather than convergence. (3)Overall, the obstacle index in Chongqing is “economic vitality > coordinated development > green development > digital development,” underscoring the critical role of economic vitality in high-quality economic development. Differences exist between the main urban area and the “two groups,” with the “two groups” following the overall pattern while the main urban area prioritizes “green development > economic vitality > coordinated development > digital development.” (4)According to the results of the analysis of the indicator level, Chongqing and the main city metropolitan area should focus on the development of the three dimensions of economic vigor, coordinated development, and green development, while the northeastern and southeastern parts of Chongqing should focus on economic vigor and coordinated development.

Based on the above research conclusions and the current situation of high-quality economic development in Chongqing, this paper draws some possible ways to enhance the future high-quality development of Chongqing’s economy: (1)Continuously Promote High-Quality Regional Economic Development: While Chongqing's "one district and two groups" have improved yearly, this trend should be further consolidated. By fostering development in all sectors, a strong synergy can push high-quality economic development to new heights. (2)Leverage the Leading Role of the Main City Metropolitan Area: As the forefront of Chongqing's high-quality economic development, the main city metropolitan area should continue enhancing its development level, providing reference experiences and models for the "two groups." The "two groups" should also leverage their advantages to accelerate development and catch up. (3)Implement Differentiated Development Strategies: Given the varying development levels across different dimensions, targeted policies and measures should promote coordinated and balanced regional development. For instance, the main city new area should boost economic vitality and digital development while strengthening coordinated and green development. Northeast Chongqing should enhance economic vitality and coordinated development. (4)Strengthen Regional Cooperation and Coordination: Regional cooperation and exchanges should be strengthened to promote the rational flow of resources, technologies, and talents. Enhancing coupling and coordination can achieve common development and mutual benefits. Government policy support should accelerate the improvement of the science and technology financial support system, fully utilizing the natural environment advantages of the "two groups" and promoting the development of advantageous industries in districts and counties. All districts and counties should deepen cooperation to share high-quality resources and complementary advantages, improving their overall economic level and narrowing regional coupling and coordination gaps. (5)Prioritize Strengthening Support for Economic Vitality, Coordinated Development, and Green Development: According to the obstacle indicators, support for economic vitality, coordinated development, and green development should be prioritized to advance the high-quality development process. Simultaneously, the importance of digital development should be recognized, with increased investment in related fields to enhance digital development. The main city metropolitan area should focus on green development support, while the "two groups" should emphasize economic vitality and coordinated development. Strengthened regional cooperation and focused development of key areas will collectively elevate the high-quality development of the regional economy.

## 4.It is recommended to review the article for grammatical errors to enhance its readability.

This article has been corrected for possible grammatical problems.

## **5.Although this article focuses on evaluating the coupling coordination degree and diagnosing obstacle factors, there is excessive description regarding the selection of indicators, which could be condensed.**

The indicator description section has been removed from this article.

(1) Economic vigor. economic vigor is an important indicator of the rate of growth of aggregate supply and demand in the economy of a country or region over a given period of time, as well as its potential. It involves not only macroeconomic indicators such as the growth rate of gross national product, the fixed investment rate and the savings rate, but also the capacity of urban economic development, such as the ability to bring in capital and attract high-quality labor. Economic vitality is closely related to high-quality economic development, because high-quality development is not only quantitative growth, but more importantly qualitative improvement, including the optimization of economic structure (Li Ling'e, 2022), the transformation and upgrading of industries (Liao Changwen, 2023), and the coordinated development of urban and rural areas (Zhang Hongfeng, 2022), among other aspects. In the process of promoting high-quality economic development, the measurement of economic vitality is particularly important. It can help us identify and solve the structural contradictions and institutional mechanism obstacles that constrain economic development, and promote economic restructuring and the transformation of old and new kinetic energy.

(2) Harmonious development. harmonious development is an important criterion for measuring high-quality economic development, this is because it embodies the comprehensiveness and balance of economic development, and ensures the coordinated progress of various fields, regions and social strata. It emphasizes improving social well-being and the ecological environment while enhancing economic indicators, promoting balanced development among different regions, industries and social groups, ensuring rational use of resources and intergenerational equity, enhancing the economy's innovative and risk-resistant capabilities, and achieving economic and social development goals through policy guidance (Ding Xueqian, 2022; Han Xu, 2023; Li Sufeng, 2024). Coordinated development helps improve the country's international competitiveness and promotes long-term stable economic growth and comprehensive social progress. In the context of the new era, China's economy has shifted from the stage of high-speed growth to the stage of high-quality development, a shift that requires the economy not only to pursue quantitative growth, but also to focus on quality improvement and structural optimization.

(3) Green development. Green development is closely related to high-quality economic development, which is not only an important content of high-quality development, but also the key to promoting economic transformation and upgrading. The core of green development is to achieve economic growth, improve resource utilization efficiency, reduce pollution emissions, and promote the virtuous cycle of socio-economic and natural ecosystems under the premise of protecting the environment (Zhang Yutong, 2024; Liu Zhixiong, 2024). At the policy level, the Chinese government has taken green development as a national strategy to promote a comprehensive green transformation of economic and social development by formulating relevant policies and standards. For example, it promotes the green and low-carbon transformation and upgrading of traditional industries, vigorously develops green and low-carbon industries, accelerates the synergistic transformation and development of digitalization and greening, and steadily promotes the green and low-carbon transformation of energy. These measures are aimed at building a green, low-carbon and recycling economic system, realizing a steady decline in carbon emissions after reaching the peak, and laying a solid foundation for building a beautiful China. In addition, green development involves the optimization of spatial patterns, such as optimizing the pattern of territorial spatial development and protection, creating green development highlands, and building a spatial pattern of green, low-carbon and high-quality development. These measures help to form green modes of production and lifestyles, improve resource utilization efficiency, and reduce environmental pollution, thus promoting high-quality economic development.

(4) Digital development. A large number of studies have shown that the digital economy can effectively promote the high-quality development of the economy (Jing Wenjun et al., 2019; Qi Yudong, 2024). Digital development can significantly improve production efficiency, promote innovation, enhance market responsiveness, and promote industrial upgrading and regional coordinated development (Ren Baoping, 2023). Digital transformation injects new momentum into economic growth by optimizing resource allocation, modernizing the industrial chain, improving the efficiency of government services, strengthening data security governance, promoting green development, enhancing international competitiveness, and facilitating the optimization of employment structure (Chao Xiaojing, 2024). It also helps to build a modernized economic system, enhance the country's global competitiveness and support the overall progress of society.

# References

1. Arrow K J, Dasgupta P, Goulder L H, et al. Sustainability and the measurement of wealth. Environment and Development Economics. 2012;17(3):317-353.

2. Yang Yongfang,Wang Qin. Research on the construction of evaluation index system for high-quality development of Chinese regional economy in the new era[J]. China Soft Science, 2024(S1): 182-190.

3. Chen Shiyi, Chen Dengke. Haze Pollution, Government Governance and High-Quality Economic Development[J]. Economic Research, 2018, 53 (02): 20 -34.

4. Liu Zhibiao, Ling Yonghui. Structural transformation, total factor productivity and high-quality development[J]. Management World, 2020, 36 (07): 15 -29.

5. Jing Wenjun, Sun Bowen. Digital economy for high-quality economic development:a theoretical analysis framework[J]. Economist, 2019,(02).

6. Zhou C, Li X, Lin X, et al. Influencing factors of the high-quality economic development in China based on LASSO model[J]. Energy reports, 2022, 8: 1055-1065.

7. 7.Yang Y, Su X, Yao S. Nexus between green finance, fintech, and high-quality economic development: Empirical evidence from China[J]. Resources Policy, 2021, 74: 102445.

8. Gao J, Wu D, Xiao Q, et al. Green finance, environmental pollution and high-quality economic development—a study based on China’s provincial panel data[J]. Environmental Science and Pollution Research, 2023, 30(11): 31954-31976.

9. Chen L, Huo C. The measurement and influencing factors of high-quality economic development in China[J]. Sustainability, 2022, 14(15): 9293.

10. Zhang W, Zhao S, Wan X, et al. Study on the effect of digital economy on high-quality economic development in China[J]. PloS one, 2021, 16(9): e0257365.

11. Guo B, Wang Y, Zhang H, et al. Impact of the digital economy on high-quality urban economic development: Evidence from Chinese cities[J]. Economic Modelling, 2023, 120: 106194.

12. Du J, Zhang J, Li X. What is the mechanism of resource dependence and high-quality economic development? An empirical test from China[J]. Sustainability, 2020, 12(19): 8144.

13. Chen L, Ye W, Huo C, et al. Environmental regulations, the industrial structure, and high-quality regional economic development: Evidence from China[J]. Land, 2020, 9(12): 517.

14. Wang J, Zhang G. Can environmental regulation improve high-quality economic development in China? The mediating effects of digital economy[J]. Sustainability, 2022, 14(19): 12143.

15. Shen W, Xia W, Li S. Dynamic coupling trajectory and spatial-temporal characteristics of high-quality economic development and the digital economy[J]. Sustainability, 2022, 14(8): 4543.

16. Changzheng Z, Jin K. Effect of equity in education on the quality of economic growth: evidence from China[J]. Journal of Human Sciences, 2010, 7(1): 47-69.

17. Hua X, Lv H, Jin X. Research on high-quality development efficiency and total factor productivity of regional economies in China[J]. Sustainability, 2021, 13(15): 8287.

18. Chen S, Chen D. Haze pollution, government governance and high-quality economic development[J]. Economic Research, 2018, 53(02): 20-34.

19. Ge H, Wu F. Digital economy enables high-quality economic development: theoretical mechanisms and empirical evidence[J]. Frontiers of Economics in China-Selected Publications from Chinese Universities, 2022, 17(4): 643-668.

20. Li Ling'e. Construction of modernized economic system and high-quality development of resource-based economy[J]. Review of Political Economy,2022,13(05):59-86.

21. Liao Changwen, Zhang Zhidong. Industrial Integration, Industrial Structure Optimization and High-Quality Economic Development-Evidence from the Yangtze River Delta City Cluster[J]. Economic System Reform,2023,(06):62-71.

22. Zhang Hongfeng, Cheng Peng. Research on the coupling coordination degree of China's urban-rural coordination level and high-quality economic development[J]. Journal of Shandong University of Finance and Economics,2022,34(04):65-76.

23. Ding Xueqian, Wu Qun, Liu Xiangnan,et al. The degree of coordination and factors influencing the coupling of land use, high-quality economic development and carbon emission-an empirical study from 282 prefecture-level cities in China[J]. Resource Science,2022,44(11):2233-2246.

24. Han Xu, Liu Xiaoming, Liu Wenting, et al. Spatial and temporal characteristics and driving factors of coupled coordination of green finance and high-quality economic development in the Yellow River Basin[J]. Economic Geography,2023,43(09):121-130.

25. Li Sufeng, Yang Lei, Feng Hongyan. Green finance, environmental regulation and high-quality economic development-A comparative analysis based on the Beijing-Tianjin-Hebei coordinated development strategy[J]. Journal of Central University of Finance and Economics,2024,(03):3-15.

26. Zhang Yutong, Zhang Songrui, He Shoukui. A study on the mechanism of environmental regulation and scientific and technological innovation synergizing to promote high-quality economic development--Taking 16 cities in the twin-city economic circle of Chengdu-Chongqing region as an example[J]. Resource Development and Market,2024,40(03):401-410.

27. Liu Zhixiong, Zhang Jiuyue, Chen Honghui. An empirical study on the impact of China's ecological environmental governance on high-quality economic development[J]. Ecological Economy,2024,40(06):172-178.

28. Qi Yudong , Du Bo. Digital economy, high-quality development and the promotion of Chinese-style modernization[J]. Journal of Shandong University (Philosophy and Social Science Edition),2024,(01):108-124.

29. Ren Baoping, Wang Ziyue. The Logic and Path of Digital New Quality Productivity to Promote High-Quality Economic Development[J]. Journal of Xiangtan University (Philosophy and Social Science Edition),2023,47(06):23-30.

30. Chao Xiaojing, Wang Chenwei , Wang Can. Theoretical Mechanism and Realization Path of Digital Economy to Promote High-Quality Economic Development[J]. Economic Journal,2024,(03):108-117.
